# Oscillatory brain responses to own names uttered by unfamiliar and familiar voices

**DOI:** 10.1016/j.brainres.2014.09.074

**Published:** 2014-12-03

**Authors:** Renata del Giudice, Julia Lechinger, Malgorzata Wislowska, Dominik P.J. Heib, Kerstin Hoedlmoser, Manuel Schabus

**Affiliations:** a University of Salzburg, Department of Psychology, Laboratory for Sleep, Cognition and Consciousness Research, Hellbrunnerstrasse 34, 5020 Salzburg, Austria; b Center for Cognitive Neuroscience Salzburg (CCNS), University of Salzburg, Hellbrunnerstrasse 34, 5020 Salzburg, Austria

**Keywords:** Oscillations, Disorders of consciousness, EEG, Subject’s own name, Familiar voice

## Abstract

Among auditory stimuli, the own name is one of the most powerful and it is able to automatically capture attention and elicit a robust electrophysiological response. The subject’s own name (SON) is preferentially processed in the right hemisphere, mainly because of its self-relevance and emotional content, together with other personally relevant information such as the voice of a familiar person. Whether emotional and self-relevant information are able to attract attention and can be, in future, introduced in clinical studies remains unclear. In the present study we used EEG and asked participants to count a target name (active condition) or to just listen to the SON or other unfamiliar names uttered by a familiar or unfamiliar voice (passive condition). Data reveals that the target name elicits a strong alpha event related desynchronization with respect to non-target names and triggers in addition a left lateralized theta synchronization as well as delta synchronization.

In the passive condition alpha desynchronization was observed for familiar voice and SON stimuli in the right hemisphere.

Altogether we speculate that participants engage additional attentional resources when counting a target name or when listening to personally relevant stimuli which is indexed by alpha desynchronization whereas left lateralized theta synchronization may be related to verbal working memory load. After validating the present protocol in healthy volunteers it is suggested to move one step further and apply the protocol to patients with disorders of consciousness in which the degree of residual cognitive processing and self-awareness is still insufficiently understood.

## Highlights

• EEG during an active–passive task based on first names was time-frequency analyzed.
• The presented names were uttered either by an unfamiliar or a familiar voice.
• Counted names elicited alpha desynchronization and left theta synchronization.
• Own name and familiar voices enhanced strong right alpha desynchronization.
• Alpha desynchronization reflects attentional engagement and emotional processing.

## 1 Introduction

Many studies have investigated auditory processing of the subject’s own name (SON). Also because of its countless repetitions during lifetime, the SON is intrinsically meaningful to individuals. In fact, among auditory stimuli, the own name is considered the most powerful stimulus which captures attention without any voluntary effort, as for example demonstrated in the classical “cocktail party” phenomenon (Holeckova et al., 2006, Mack et al., 2002, Moray, 1959), or by its residual processing during non-conscious states such as sleep (Perrin et al., 1999, Portas et al., 2000).

EEG studies have shown that the presentation of the SON evokes larger “P300” (Berlad and Pratt, 1995) or “P3” responses (Folmer and Yingling, 1997) than other first names, which is to be expected, as the P3 is the most significant event-related potential that is known to be related to the processing of relevant or “target” stimuli (Donchin and Cohen, 1967). In the frequency domain, only recently responses to SON have been studied. It has been reported that alpha (8–12 Hz) and theta (4–7 Hz) activity reflect attentional and/or memory processes (Fingelkurts et al., 2002, Klimesch, 1999, Klimesch, 2012). The evaluation of on-going oscillatory activity in response to SON stimuli can therefore shed light on involved cognitive functions. With respect to event-related response Tamura et al. (2012) found stronger theta event-related synchronization (ERS) to the SON which they interpreted as attentional engagement. Other recent studies found a decrease in alpha power in response to SON presentation which the authors likewise interpreted in terms of enhanced alertness or increased active processing due to release of inhibition (Höller et al., 2011, Ruby et al., 2013). Interestingly, also in patients suffering from a disorder of consciousness (DOC) or locked in syndrome (LIS) it is known that the salient SON can still evoke a significant brain response. Surprisingly not only minimally conscious state (MCS) but even supposedly unaware vegetative state/unresponsive wakefulness syndrome (VS/UWS) patients (Perrin et al., 2006) seem to be able to differentiate their own name from other names. A similar study by Fischer in line with these findings reports that some DOC patients, irrespective of their diagnosis, are able to process SON stimuli when they are presented as deviant stimuli in a stream of tones. The authors suggest that the processing of stimulus novelty might prove preservation of some cognitive function independent of conscious awareness (Fischer et al., 2010).

Because of its self-relevance and its emotional content, the SON is preferentially processed in the right hemisphere together with other personally relevant information (Adolphs et al., 1996, Perrin et al., 2005; Schwartz et al., 1975; Van Lancker and Klein, 1990). More interestingly, the activation of the right temporal-parietal junction in response to SON has been related to self-recognition processes (Holeckova et al., 2008). Interestingly, the processing of familiar voices or identifying the individual identity of voices likewise elicits right hemispheric dominant brain responses (Levy et al., 2001, Nakamura et al., 2001).

However, it has been discussed that the passive own name paradigm, in which subjects only passively listen to the presented stimuli might reflect mere automatic stimulus identification and does not allow for an inference about the level of preserved awareness (Bruno et al., 2011, Davis et al., 2007). Addressing this criticism, several EEG studies instructed participants and patients to focus their attention on an auditory target stimulus while ignoring other irrelevant stimuli (Schnakers et al., 2009a, Schnakers et al., 2008). Specifically, a greater P3 component for attended stimuli was observed in controls as well as in MCS patients (Schnakers et al., 2008). In a more recent study using time–frequency analysis, greater alpha event related desynchronization (ERD) was evident when participants were asked to count the SON, probably reflecting enhanced attentional engagement (Fellinger et al., 2011). In addition, stronger theta event related synchronization (ERS) reflecting working memory involvement was found when subjects were counting as compared to listening to the SON. This task related theta-synchronization was only evident for the SON, but not for unfamiliar name (UN) stimuli, indicating that top-down processes might be easier to engage when the stimulus is emotionally salient and already strongly bottom-up processed. In line with this view, it has been demonstrated earlier that familiar objects, because of their biographical and emotional relevance, are able to increase the number of responses as well as their goal-directedness in DOC patients (Di Stefano et al., 2012). Furthermore, meaningful stimuli with high emotional valence, such as infant cries or the voice of a family member, can induce more widespread “higher-order” cortical responses (Bekinschtein et al., 2004, Di et al., 2007, Jones et al., 1994, Laureys et al., 2004) and facilitate applying top-down attention to relevant input (de Jong et al., 1997, Fellinger et al., 2011, Holeckova et al., 2006). Given those findings, we believe that it is important to further elaborate on study protocols which focus on emotionally relevant stimuli on an individual level.

In the current study we used a modified version of the classical own name paradigm including an active “counting” as well as a familiar voice condition. The active condition, in which subjects were asked to (silently) count a specific unfamiliar name should give an important insight in the amount of top-down control and attentional resources engaged by target names and could, therefore, in future studies allow for identifying “awareness” in patients suffering from DOC, in whom behavioural assessment is often challenging and leads to high rates of misdiagnosis (Andrews et al., 1996, Schnakers et al., 2009b). The introduction of familiar voices aims at increasing the bottom-up stimulus strength by adding emotional valence, which should make it easier to attend to the presented stimuli and will provide us with important information regarding the processing of emotional and self-relevant information in the absence of an explicit cognitive demand.

We will focus on on-going oscillatory activity that is not necessarily exactly time-locked to the presentation of the stimulus, like event-related potentials. In fact, time–frequency analysis, quantifying evoked as well as induced brain activity, has been shown to be more sensitive than mere evoked responses which are more prone to temporal dispersion (Mouraux and Iannetti, 2008). Furthermore, concerning the intended clinical application in DOC patients in the future, it is important to consider that many DOC patients have prevailing background activity in the delta range that can interfere substantially with event-related potentials (Kotchoubey et al., 2005, Neumann and Kotchoubey, 2004, Sabri and Campbell, 2002). Consequently, we believe that using time-frequency analysis together with a modified own name paradigm using emotionally and personally salient stimuli will be a more sensitive measure in identifying cognitive, and in future clinical applications, conscious processing.

## 2 Results

### 2.1 Alpha ERD in the active counting condition

The main findings of ANOVA CONDITION (target vs. non-target; both spoken in a familiar voice)×ELECTRODES (Fz vs. Cz vs. Pz)×TIME (*t*1 vs. *t*2 vs. *t*3 vs. *t*4; *t*1=0–200 ms, *t*2 =200–400 ms, *t*3=400–600 and *t*4=600–800 ms post-stimulus),) showed that alpha desynchronization was higher for the target than for non-targets (*F*_1/13_=5.98, *p*<.05) (cf. Fig. 2, Fig. 3).

Additionally, main effects for ELECTRODES (*F*_2/26_=5.46, *p*<.05) and TIME (*F*_3/39_=8.05, *p*<.001) were revealed. Post hoc tests revealed that *t*3 and *t*4 significantly differed from *t*1 (*t*(13)=−3.88, *p*<.05; *t*(13)=−3.18, *p*<.05) while *t*3 differed from *t*2 (*t*(13)=−3.55, *p*<.05). Furthermore, alpha ERD was higher on the electrode Pz compared to Cz (*t*(13)=2.86, *p*<.05) indicating generally larger desynchronization in the posterior part of the scalp and in particular in the last two time windows.

The difference between the two conditions is also embedded in the interactions CONDITION×ELECTRODES (*F*_2/26_=5.27, *p*<.05) and CONDITION×TIME (*F*_3/39_=11.44, *p*<.001). Post-hoc tests on the first interaction revealed that target stimuli evoke stronger alpha ERD compared to non-targets mainly over Pz (*t*(13)=2.51, *p*=0.013) while post-hoc testing of the latter indicated that alpha ERD was stronger in response to targets as compared to non-targets only in the later time windows (*t*3: *t*(13)=−2.47, *p*<.05; *t*4: *t*(13)=−4.32, *p*<0.001).

On the single subject level, we conducted one-sample *t* tests against zero for trials across different condition (for details see Supplementary Table 1) and found target related alpha ERD to be evident in 81% of the subjects. For an overview of event-related potentials in the active condition please also refer to supplementary material and Supplementary Fig. 1.

### 2.2 Theta ERS in the active counting condition

Theta ERS analysis revealed main effects for ELECTRODES (*F*_2/26_=32.43, *p*<.001) and TIME (*F*_3/39_=6.13, *p*<.05) as well as an interaction between ELECTRODES and TIME (*F*_6/78_=3.68, *p*<.05). According to post-hoc analyses electrodes Fz and Cz exhibited higher theta ERS as compared to the electrode Pz (*t*(13)=5.29, *p*<.001; *t*(13)=10.49, *p*<.001, respectively) indicating that theta ERS was most pronounced over fronto-central sites. Theta ERS was strongest 200–400 ms after stimulus onset followed by a steady decrease over time (*t*2>*t*3: t(13)= 3.50, *p*<.05; *t*2>*t*4: *t*(13)=3.36, *p*<.05), In addition, the interaction ELECTRODES×TIME indicated that theta ERS was systematically higher on Fz (*t*1: *t*(13)=9.45, *p*<.001; *t*2: *t*(13)=9.44, *p*<0.01; *t*3: *t*(13)=8.39, *p*<.001; *t*4: *t*(13)=5.65, *p*<0.001) and Cz in all time windows as compared to Pz (*t*1: *t*(13)=4.76, *p*<.001; *t*2: *t*(13)=6.07, *p*<0.00; *t*3: *t*(13)=5.84, *p*<.001; *t*4: *t*(13)=3.43, *p*<0.05). Results are also depicted in Fig. 3 using topography maps.

Since lateralization effects were evident for theta in the active counting condition we decided to also focus on potential hemispheric differences. An ANOVA including the factors CONDITION (target vs. non target), HEMISPHERE (C3 vs. C4) and TIME for the theta frequency revealed a nearly significant main effect for HEMISPHERE (*F*_1/12_=4.52, *p*=.055) indicating generally higher theta ERS in the left hemisphere (21.99% theta ERS on C3 vs. 18.52% at C4; *t*(12)=2.12). The interaction CONDITION×HEMISPHERE×TIME (*F*_3/36_=3.72, *p*<.05) indicated that theta ERS is greater for targets as compared to non-target on the left side of the scalp and in the time window from 200 to 400 ms (*t*(12)=2.186, *p*<.05).

On a single subject-level theta ERS was evident in more than 90% of the subjects (100% for the target condition and 92% for the non-target), as revealed by one-sample *t* tests against zero for trials across different condition (for details refer to Supplementary Table 1). Results are also depicted in Fig. 2 in time–frequency plots and across the scalp using topography maps (cf. Fig. 3).

### 2.3 Delta enhancement in the active condition

Since visual inspection of other frequency bands indicated a possible involvement of the delta band in the active condition we also tested whether there was a stimulus specific modulation in this frequency range. Surprisingly, we found a significant effect in the active condition also in the delta range. As illustrated by the main effect CONDITION (*F*_1/13_=12.16, *p*<.05) delta activity was significantly higher for target names as compared to non-targets (*t*(13)=3.48, *p*<.005) over all electrodes (Fz, Cz, Pz). Additionally, the main effect TIME (*F*_3/39_=31.22, *p*<.001) indicated that delta was modulated over time with higher ERS from 200 to 600 ms after stimulus onset (*t*2>*t*1: *t*(13)=8.98, *p*<.001; *t*3>*t*1: *t*(13)=5.65, *p*<.001; *t*2>*t*4: *t*(13)=6.01, *p*<.001; *t*3>*t*4: *t*(13)=10.17, *p*<.001). (cf. Fig. 2.)

### 2.4 Alpha ERD in the passive listening condition

Concerning the ANOVA NAME (SON vs. UN)×VOICE (FV vs. UV)×ELECTRODES (Fz vs. Cz vs. Pz)×TIME (*t*1 vs. *t*2 vs. *t*3; *t*1=0–200 ms, *t*2=200–400 ms, *t*3=400–600 ms post-stimulus) for alpha ERD during passive listening, only a main effect for TIME (*F*_2/26_=5.71 *p*<.05) was significant. Post hoc tests revealed higher desynchronization in the alpha band around 400–600 ms (*t*3) as compared to 0–200 ms (*t*1) after stimulus onset (*t*(13)=−2.82, *p*<.05). To again test for hemispheric differences, an additional ANOVA including the factors NAME (SON vs. UN), VOICE (familiar voice vs. unfamiliar voice), HEMISPHERE (P3 vs. P4) and TIME (*t*1, *t*2, *t*3) was calculated. A significant interaction VOICE x HEMISPHERE (*F*_1/13_=5.81, *p*<.05) indicated that the right parietal electrode (P4) showed higher alpha ERD for stimuli spoken in a familiar voice as compared to stimuli spoken in an unfamiliar voice (*t*(13)=−3.58, *p*<.05). In addition, the SON as compared to UN also showed enhanced alpha ERD (NAME×HEMISPHERE×TIME: *F*_2/26_=3.80, *p*<.05) over the right parietal region in the last two time windows (from 200 to 400 and from 400 to 600 ms) irrespective of VOICE (*t*(13)=−2.25, *p*<.05, *t*(13)=−2.59, *p*<.05; respectively) (cf. Fig. 4 for time–frequency plot and scalp distribution). For the respective comparisons using event-related potentials please refer to Supplementary Fig. 1.

### 2.5 Theta ERS in the passive listening condition

The ANOVA NAME×VOICE×ELECTRODES×TIME (for the factor levels please refer to 2.4) for theta frequency yielded main effects for ELECTRODE (*F*_2/26_=22.52, *p*<.001) and TIME (*F*_2/26_=5.27, *p*<.05).

Post hoc tests revealed that the electrode Pz showed less theta ERS than both Cz and Fz (*t*(13)=−5.87, *p*<.001; *t*(13)=−4.74, *p*<.001, respectively) and that theta synchronization was strongest 200–400 ms post-stimulus (*t*2) (*t*2>*t*1: *t*(13)=3.16, *p*<.05; *t*2>*t*3: *t*(13)=3.60, *p*<.05).

The topographical distribution of theta ERS for the passive condition is also depicted in Supplementary Fig. 2. For an overview of event-related potentials in the passive condition please refer to the supplementary material (Supplementary Fig. 1).

## 3 Discussion

The present study focused on oscillatory brain responses to auditory name stimuli uttered by a familiar or unfamiliar voice. In the active condition, in which subjects had to count a specific target name, a higher desynchronization in the alpha band (8–12 Hz) to target as compared to non-target stimuli was found. The response was localized around central and posterior sites and reached its maximum about 400–600 ms post-stimulus. This is coherent with previous findings showing that alpha desynchronization reflects general task demands including attentional processes (Klimesch, 1999). Considering that in our active condition subjects had to match the memorized target name to the heard name item-per-item, the result could also indicate a release of inhibition after successful matching (Klimesch, 2012).

Also left-lateralized theta (4–7 Hz) ERS in the active condition appeared to be higher for target than non-target stimuli. Since we controlled for names of relatives and friends, in the active condition only stimuli of comparable familiarity were involved and hence familiarity cannot account for the differences between targets and non-targets. The presentation of strictly unfamiliar names in the active condition in the current study allowed for a better differentiation of top-down attention, (i.e. instruction following and counting) from automatic attention which may be grabbed automatically by the presentation of the own name (Wood and Cowan, 1995). The increased theta ERS for targets on the left side is, therefore, most likely related to top-down attention and the active counting of the target name. Attending to a target name and inhibiting irrelevant name stimuli engages selective attention mechanisms and challenges working memory resources. Higher theta ERS in the left hemisphere probably reflects attention to the processing of the new information or enhanced verbal working memory engagement (Chein et al., 2003, Smith and Jonides, 1997, Smith et al., 1996).

In the active condition we also found a significant effect in the delta range (1–4 Hz), with delta showing higher synchronization for target than for non-target stimuli. Previous studies reported that in tasks where internal concentration is required in order to focus attention on a specific stimulus delta increases (Fernández et al., 1995, Harmony et al., 1996). In addition a reciprocal relationship between alpha and delta activity has been shown, in the sense that both frequencies together may contribute to inhibitory control (Knyazev, 2007). Therefore, in our study, delta increase during counting, together with alpha desynchronization, might reflect inhibition of irrelevant information (other names) and disinhibition of relevant information in order to focus attention exclusively on the target name. The active condition, as proposed in the present study might be a promising method to assess DOC and allow refinement of their diagnosis. However, it has to be mentioned that active paradigms of that kind will only be able to distinguish DOC patients at the higher end of the DOC spectrum as they require the integrity of several sensory and cognitive processes at the same time. For a future application in DOC, it would be important, however, to further examine slow oscillatory (delta–theta) band involvement, since the EEG of DOC patients is usually characterized by a predominance of slow frequencies (mainly in the delta range).

With the passive condition, we investigated differences between the processing of the subject’s own name as compared to unfamiliar names and additionally, we were interested in the differential activation in response to familiar and unfamiliar voices. In fact, in the right hemispheric parietal alpha desynchronization was higher in response to the SON as well as in response to familiar voices. Personally relevant information is known to be more powerful in attracting involuntary attention, as demonstrated by the increase in brain activity after self-referential sound presentation (Roye et al., 2007, Roye et al., 2010). As shown by Fellinger et al. (2011) in a similar paradigm, alpha ERD can be triggered by the retrieval of information stored in long-term memory (LTM) – with the LTM retrieval being a prerequisite for the identification of personal relevance – and has been interpreted as reflecting access to LTM traces that are reactivated during the on-going task (Klimesch et al., 2007). In addition, speech perception is facilitated when a highly familiar voice is presented suggesting that familiarity may even help listeners to compensate for sensory or cognitive decline (Johnsrude et al., 2013).

Concerning the found lateralization effect, the right hemispheric dominance for the SON is again possibly related to its emotional and personal relevance (Adolphs et al., 1996, Keenan et al., 2000; Schwartz et al., 1975), which is in line with the idea that top-down involvement is more strongly reflected in the right hemisphere when listening to relevant familiar sounds (Roye et al., 2010). The right lateralization of alpha ERD in response to familiar voices is also coherent with previous studies showing that the right entorhinal cortex and the anterior part of the right temporal lobe are more active during discrimination of familiar voices than during a control discrimination task (Nakamura et al., 2001). Converging evidences from fMRI studies also revealed that the right anterior superior-temporal sulcus and part of the right precuneus (Belin and Zatorre, 2003, Belin et al., 2004, Kriegstein and Giraud, 2004, von Kriegstein et al., 2003) are specifically involved in familiar voice recognition.

Additional support for a right dominance in the processing of familiar voices come from lesion studies suggesting that an impairment recognizing familiar voices (phonanosia) is only evident in cases of damage to the right hemisphere, or more specifically right temporal lobe (Lancker et al., 1989, Van Lancker and Kreiman, 1987, Van Lancker et al., 1988). Thus, there is clear converging evidence for an important role of the right hemisphere in processing voice identity.

According to the cognitive model of voice perception by Belin et al., 2011, Belin et al., 2004), following a low-level analysis in the primary auditory cortex, vocal information is processed at three interacting but partially dissociable pathways: (i) analysis of speech information, preferentially in the left hemisphere, (ii) analysis of vocal affective information, predominantly in the right hemisphere, (iii) analysis of vocal identity, involving voice recognition and person-related semantic knowledge, also predominant in the right hemisphere. In this view, different levels of cognition and awareness might be required to move from low-level to higher levels analysis. The pronounced alpha ERD for familiar voices could, therefore, indicate processing at least at the vocal affective level and might, thus, serve as marker in cases where verbal report cannot be obtained. For a potential application in DOC, the understanding of whether and to what extent patients are able to process vocal information would help to better comprehend their residual capabilities.

Since SON and FV stimuli in our study were simply presented to participants without further instruction to elaborate on them, we cannot be sure whether the right hemisphere enhancement for these “emotional” stimuli (i.e. FV and SON) is purely automatic or rather reflects higher levels of processing and emotional self-awareness. On an individual subject level in the active counting condition data revealed that 81% of participants did show alpha ERD (or 100% theta ERS), but only 64% more than to the non-target (62% for theta ERS) (cf. Supplementary Table 1). It, therefore, appears that salient information of the chosen kind is reliably evoking event-related brain responses. Introducing emotional or self-relevant information might, therefore, be a way to effectively enhance arousal and increase bottom-up stimulus processing (as demonstrated by higher theta ERS and right alpha ERD in the passive condition) which in turn might allow for the engagement of top-down processes in the first place. If the reliability of these effects is, however, sufficient for sensitive detection of residual capabilities in DOC patients has to be assessed in future studies. Experiments in healthy individuals introducing distracting material and systematically varying working memory demands could reveal whether emotional or self-relevant stimuli might still be reliably (top-down) processed in situations where limited attentional capacity usually precludes instruction following.

Furthermore, while the predominant role of the right hemisphere in the processing of self-relevant and emotional information (FV and SON) is already validated (Adolphs et al., 1996; Schwartz et al., 1975; Perrin et al., 2005), the link to self-awareness remains elusive.

In both conditions the differential contribution of alpha and theta was mirrored in the differential topographical distribution in these two frequency bands. In fact, while in the active condition alpha is more pronounced on the parietal area around the midline theta is higher over left central regions. In the passive condition alpha is right lateralized. These differences in scalp distribution might, therefore, underline the involvement of different cerebral structures and source localization studies should further elucidate that.

In summary, our results demonstrate that time frequency analysis allows for studying the correlates of an active task demand in combination with voice familiarity. Alpha ERD seems to reflect the release of inhibition after successful memory matching. In addition, theta ERS is pronounced when selective attention is attracted by personally relevant information and when incoming information matches long term memory representations, such as a familiar voice or a subject’s own name. Ultimately, we hope that this paper will stimulate new perspectives in order to access and assess (self) awareness also in clinical populations such as DOC patients.

## 4 Experimental procedures

### 4.1 Subjects

A sample consisting of 14 subjects (9 females, 5 males) with age ranging from 21 to 53 (*M*=25.79; SD=8.17) was recorded. All volunteers were right-handed German native speakers without any recorded history of neurological disease. Participants gave written informed consent approved by the local ethics committee and received monetary compensation for their participation.

### 4.2 Experimental design and procedure

The experiment expands the SON task as introduced by Schnakers et al. (2008) and subsequently adapted in Fellinger et al. (2011). Stimuli were either spoken by a familiar (FV; subject’s close friend or family member) or unfamiliar voice (UFV; spoken by a text-to-speech algorithm, CereProc^®^, CareProc Ltd: “Alex”, “Gudrun”). Stimuli included the subject’s own name and five commonly used Austrian names (according to statistics Austria) matched for number of syllables and the gender of the participant. Stimuli were presented via headphones at a sound pressure level of 80 db.

**Fig. 1:**
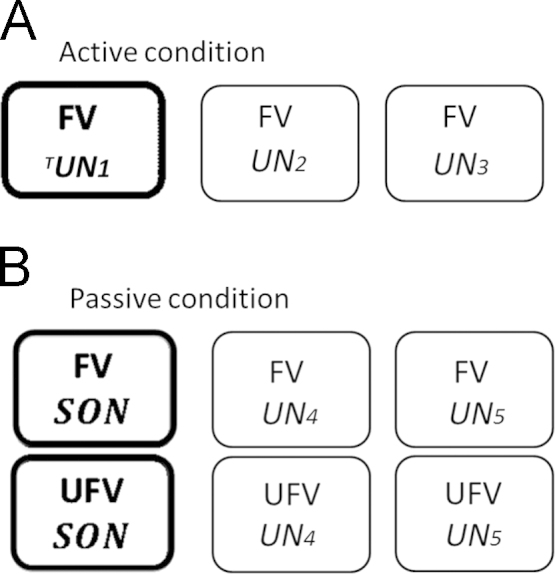
Own name task design using familiar voice manipulation. (A) The active condition consisted of only three different unfamiliar names (UN_2_, UN_3_) with one name being the attended target name (^T^UN_1_). (B) The passive condition consisted of six different stimuli which participants attended to. Own names and unfamiliar names and both uttered in a familiar or unfamiliar voice. Abbreviations: Familiar voice [FV]; unfamiliar voice [UFV]; subject’s own name [SON]; unfamiliar name [UN].

The task consisted of two experimental conditions: an active condition to investigate the ability to consciously follow commands and a passive listening condition with the passive condition always preceding the active condition. Each condition consisted of 3 blocks; with each block including 13 presentations of each name (i.e., 39 presentations for each single name). In the passive condition 6 stimuli were presented with 234 repetitions in total (about 12 min), in particular, SON uttered by a familiar or unfamiliar voice and two different unfamiliar names either spoken by a familiar or an unfamiliar voice. In the active condition only 3 different stimuli were presented (117 repetition) for about 6 min, all of them unfamiliar to participants and all uttered by a familiar voice (cf. Fig. 1). During the passive condition participants were simply asked to listen to all the names presented, while in the active condition they were asked to focus and silently count the appearance of the target name. In order to be sure that participants attended the presented stimuli experimenters controlled at the end of the experiment whether the number of targets counted by participants matched the total number of stimuli presented and controlled online for arousal fluctuations. The inter stimulus interval [ISI] lasted 2000 ms and for stimulus presentation and synchronization, the Software Presentation^®^, (Version 0.71; Presentation Software, Neurobehavioralsystems Inc., CA) was used.

**Fig. 2:**
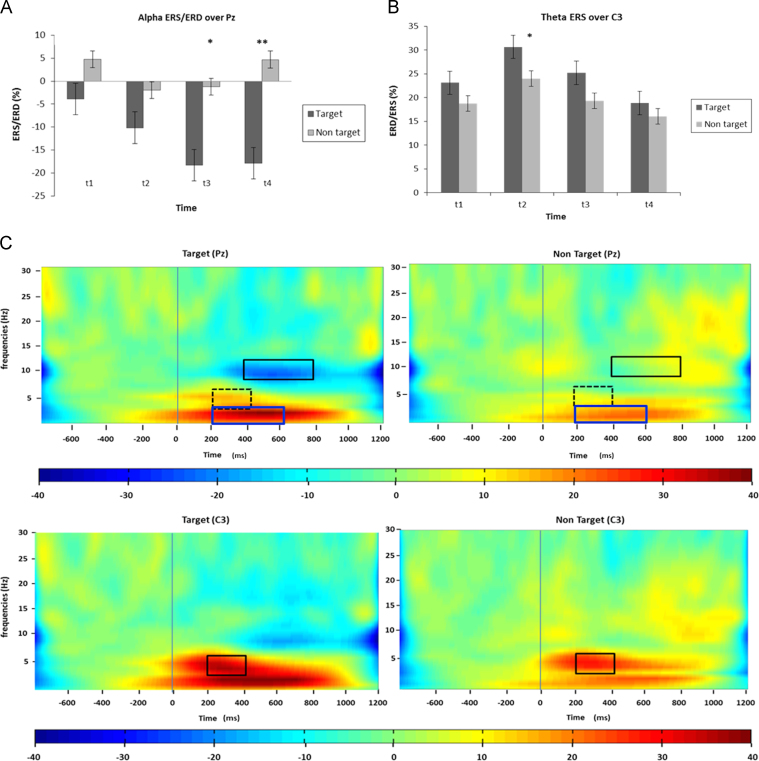
ERS/ERD during the active counting condition. (A) The graph depicts the mean (0–800 ms) of alpha ERD for targets and non-targets over electrode Pz. (B) Theta ERS for targets and non-targets over left-central site C3. Error bars represent ±1 standard error of mean, asterisks denote the respective significance-level for post hoc comparisons: *p<.05, **p<.01. (C) Time frequency plots depict stronger delta ERS and alpha ERD in the target than non-target condition at parietal electrodes (Pz, upper panel) and stronger theta ERS at left-central (C3, lower panel) electrode sites. Zero marks the presentation of the stimuli, with solid rectangles (black for alpha and blue for delta) in the plot highlighting significant differences between targets and non-targets and dashed lines indicating trends for theta ERS. Time windows: *t*1=0–200 ms, *t*2=200–400 ms, *t*3=400–600 and *t*4=600–800 ms post-stimulus.

**Fig. 3:**
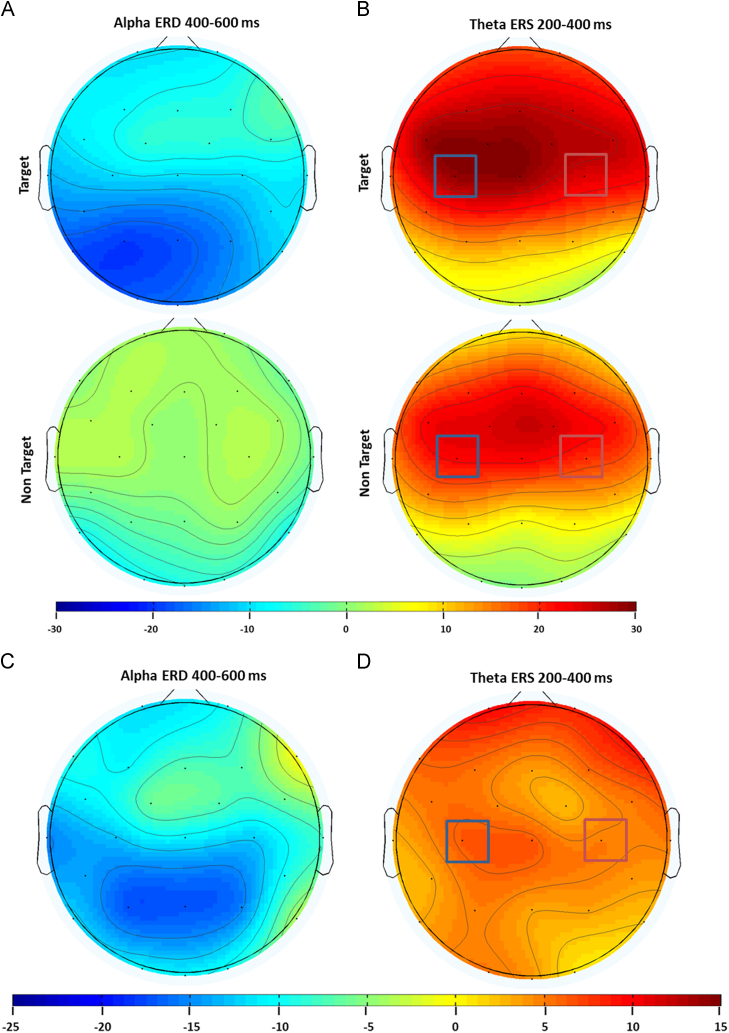
(A) Topographic maps depict the topographic distribution for alpha ERD (400–600 ms) and (B) theta ERS (200–400 ms) in the active condition. (C) Panel C depicts the topographic distribution of the difference between targets and non-targets for alpha ERD. (D) Panel D shows the topographical distribution of the difference between targets and non-targets for theta ERS. Note that alpha ERD is more pronounced for targets than for non-targets in the central and posterior part of the scalp (left panel) while theta ERS is higher for targets than non-targets in the frontal and central portion of the scalp. Squares indicate electrodes where hemispheric asymmetry was modulated by target and non-target stimuli.

**Fig. 4:**
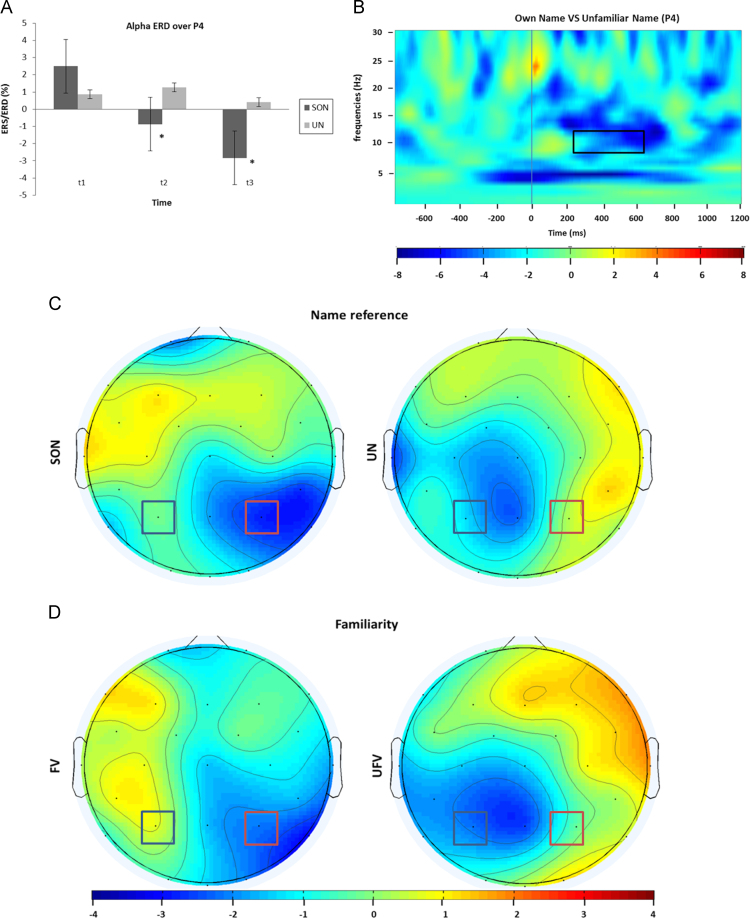
Alpha ERD during the passive listening condition. Upper panel: (A) Graph depict alpha ERD for own name and unfamiliar names over the right parietal electrode (P4), which is higher from 200 to 600 ms post-stimulus. Error bars represent ±1 standard errors of the mean, and asterisks (*) denote the respective significance-level for post hoc comparisons (*p<.05). Time windows: *t*1=0–200 ms, *t*2=200–400 ms and *t*3=400–600 ms post-stimulus. (B) Time frequency plots depict the difference between own name and unfamiliar name at right parietal (P4) electrode sites indicating stronger alpha ERD for own names compared to unfamiliar. Zero marks the presentation of the stimuli, with solid rectangles in the plot highlighting significant differences between conditions. Lower panel: Topographic maps of alpha ERD in the passive condition (400–600 ms). (C) Note that own vs. unfamiliar name presentation leads to a stronger alpha ERD over right posterior portion of the scalp. (D) Likewise, stimuli uttered by a familiar vs. unfamiliar voice evoke stronger alpha ERD over the right parietal region.

### 4.3 Data acquisition

EEG was recorded with 32 Ag/AgCl sintered electrodes and head circumference matched Easycaps (EASYCAP GmbH; Herrsching Germany) placed according to the international 10–20 system. The following scalp EEG channels were used: FP1, FP2, F7, F3, FC5, FC1, Fz, F4, F8, FC2, FC6, T7, C3, Cz, C4, T8, CP5, CP6, P7, P3, Pz, P4, P8, O1, Oz, O2, AFz and FCz. Additional electrodes were placed on the left and right mastoids (M1 and M2). For electrooculography (EOG) two horizontal (placed at the outer canthus of each eye) and two vertical (placed above and below the right eye) electrodes were used for latter correction of blinks and saccadic eye movements.

Electrodes were placed on the scalp by applying abrasive electrolyte gel, preceded by a gentle peeling (NuprepTM, Weaver and Company) and on the face secured with plasters.

EEG was recorded with a 32-channel BrainAmp EEG amplifier (Brain Products GmbH, Gilching, Germany) and Brain Vision Recorder (Brain Products). The EEG sampling rate was set to 500 Hz. Impedances were kept below 5 kΩ. AFz electrode served as a ground electrode while FCz was the recording reference electrode; the mastoid electrodes, M1 and M2 were used for later re-referencing.

Acoustic stimuli were delivered binaurally over headphones and surrounding noise was reduced to a minimum.

### 4.4 Data analysis

In a first step data was re-referenced to mastoids and bandpass—filtered between 0.5 and 70 Hz, a notch filter was set to 50 Hz. Ocular correction was conducted using the regression-based approach (Gratton et al., 1983) implemented in Brain Vision Analyzer 2.0 (Brain Products, Gilching, Germany). Afterwards, data was visually checked for further artefacts and only artefact free trials were used for analysis. Then data was segmented into epochs ranging from −800 to +1200 ms relative to stimulus-onset. For time–frequency spectral analyses, complex Morlet wavelet transformations as implemented in Brain Vision Analyser 2.0 (Brain Products, Gilching, Germany) were applied. We calculated wavelet coefficients for frequencies between 1 and 30 Hz (Morlet parameter *c*=8, linear frequency steps) with 30 frequency steps. Subsequently the wavelets were averaged across each stimulus type.

After wavelet transformation all epochs were averaged together for each participant, each condition and each stimulus type separately. In order to have comparable amounts of segments to be compared, non-target stimuli (^FV^UN2/^FV^UN3) in the active condition and unfamiliar names (^FV^UN4/^FV^UN5 and ^UFV^UN4/^UFV^UN5) in the passive condition were averaged together and only 50% of artefact free segments were randomly selected for further analysis.

### 4.5 ERD/ERS

For statistical analysis we selected two frequency bands of interests: theta and alpha in order to estimate whether presented stimuli were able to trigger attention and memory processes. For the above mentioned frequencies we chose well-established frequency ranges (Klimesch, 1999) (4–7 Hz for theta and 8–12 Hz for alpha; frequency borders: from 3.58 to 7.73 Hz for theta and 7.17 to 13.25 Hz for alpha) and concentrated on midline electrodes (Fz, Cz, Pz). For delta frequency we selected the frequency range from 1 to 4 Hz (filter borders: 0.90–4.42 Hz) (Niedermeyer and da Silva, 2005). With the obtained wavelet coefficients we calculated event related de-/synchronization, reflecting the percentage change in test power with respect to a reference interval (Pfurtscheller and Aranibar, 1977) according to the formula: ERD%=[(test−reference power)/reference power]×100. Note that contrary to the original formula we express ERS with positive and ERD with negative values. As a reference period, the time period between −700 and −200 ms relative to stimulus onset was used.

### 4.6 Statistical analysis

Five different repeated measures ANOVAs were calculated, four with theta and alpha ERS/ERD as dependent measures and one with delta ERS. Three ANOVAs tested for effects in the active condition and focused on alpha, delta and theta ERS/ERD as dependent variables, respectively: CONDITION (target, non-target), TIME (*t*1, *t*2, *t*3, *t*4; *t*1=0–200 ms, *t*2=200–400 ms, *t*3=400–600 and *t*4=600–800 ms post-stimulus), ELECTRODES (Fz, Cz, Pz). For elimination of multiple comparisons error the false discovery rate (FDR) correction according to Benjamini and Hochberg (2000) was used. Two ANOVAs were performed in order to test the effect of familiar and unfamiliar voices on stimulus processing in the passive condition: NAME (SON vs. UN), VOICE (FV vs. UV), ELECTRODES (Fz, Cz and Pz) and TIME (*t*1, *t*2, *t*3; *t*1=0–200 ms, *t*2=200–400 ms, *t*3=400–600 ms post-stimulus). Additional ANOVAs were performed post-hoc in order to specify hemispheric asymmetries apparent in the passive listening and active counting condition. For post-hoc tests we only focus on effects of interest, that is interactions with factor TARGET for the active condition and factors VOICE and NAME for the passive.

ERPs results for all conditions are also reported in supplementary materials as well as individual ERS/ERD values, tested against zero, for the active condition.

All the mentioned analyses were conducted on a sample of 14 healthy volunteers except the ANOVA to test specific hemispheric asymmetry in the processing of target, which was calculated with 13 subjects due to an outlier (power exceeding M±2 SD on C3 and C4).
