# Supplementary material for: Oscillatory brain responses to own names uttered by unfamiliar and familiar voices
Source: Brain Res. 2014 Dec 3;1591:63–73. doi: 10.1016/j.brainres.2014.09.074 (PMC4235780; doi:10.1016/j.brainres.2014.09.074)
Supplement: Supplementary file 1 — Supplementary material [file mmc1.docx]

**Event-related potentials in the ACTIVE counting condition**

The P300 component was determined for individual averages (in the time window from 200 to 400 ms after stimulus onset) according to maximum amplitude. The first ANOVA including factors CONDITION (Target vs Non-target) and ELECTRODES (Fz, Cz and Pz) revealed only a main effect for ELECTRODES (F_2/26_ = 16.04, p < .001) indicating higher P300 amplitude over the frontal and central part of the scalp as compared to electrode Pz wherein the evoked response is in the negative range (Fz > Pz, t = 4.55, p = .001; Cz > Pz, t = 5.61, p < .001).

Since oscillatory analyses of the active condition revealed a difference between target and non-target in the alpha band at 400 and 600 ms post stimulus, we decided to statistically test whether the event-related components differed in the same time window between the two conditions. The ANOVA again only revealed a main effect for ELECTRODES (F_2/26_ = 10.68, p < .001) indicating that event-related responses in this time window were in general stronger at the posterior part of the scalp (Fz < Pz, t = - 3.48, p = .004; Cz < Pz, t = -4.21, p = .001). This fits well with the posterior localization of the described alpha effect. As illustrated in Suppl. Fig. 1A target stimuli visually evoked a more positive response than non-targets in the time window from 400 to 600 ms but neither the main-effect nor the interaction with the factor CONDITION reached significance.

**Event-related potentials in the PASSIVE listening condition**

For the passive condition P300 amplitudes were subjected to an ANOVA ELECTRODES (Fz, Cz and Pz) x NAME (SON vs UN) x VOICE (FV vs UFV). Only the main effect for ELECTRODES turned out significant (F_2/26_ = 18.51, p < .001), revealing a more positive deflection over electrodes Fz and Cz as compared to Pz (Fz > Pz, t = 4.55, p = .001; Cz > Pz, t = 6.33, p = .000) which again is in line with the scalp distribution of the theta modulation in the passive condition (cf. Suppl. Fig.2). Even though Suppl. Fig. 1B shows a slightly higher positive peak in response to SON as compared to UN (and delayed in time with respect to the principal P300 component) neither factor NAME or VOICE revealed a significant effect.

Note that in the passive condition the alpha band was modulated by voice and names in a later time window (from 400 to 600 ms) over electrodes P4 and P3. Therefore we decided to test whether in the same time window ERPs were also modulated by the familiarity of the presented stimuli. The ANOVA revealed a significant main effect for VOICE (F_1/13_ = 14.88, p < .05), indicating that, in the time window from 400 to 600 ms after stimulus, ERP positivity was more pronounced for FV than UFV (t = 3.85, p = .002) (cf. Suppl. Fig. 1C). This result is again in line with our oscillatory results (cf. Fig. 5) where alpha desynchronization was higher for FV than UFV, at the right-posterior site (P4).

<insert suppl. Fig. 1 about here>

**Conclusions**

In the active condition event-related responses were not able to differentiate between targets and non-targets although visually an increased positivity was evident 400-600 ms. Also in the passive condition the P300 component was not statistically altered by our NAME and VOICE manipulation. Nevertheless, a positive component from 400 to 600 ms was specific for the familiarity of the voice and this is in line with time-frequency analysis revealing an alpha ERD difference 400-600ms.

Finally the ERP scalp distribution, as revealed by the main effects for ELECTRODES in both the active and the passive condition, is mirroring the topographical distribution of our ERS/ERD results with strongest theta ERS / alpha ERD over frontal and central electrodes and posterior sites modulated by the effect of voice in the later time window.

<insert suppl. Tab. 1 about here>

Supplementary Figure 1: Grand-averaged event-related potentials in active (A) and passive (B - C) condition.

(A) Event-related potentials in the active counting condition over electrode Fz, Cz, and Pz. Zero marks the presentation of the stimuli, with solid line representing targets and dashed line representing non-target stimuli. (B - C) Event –related potentials in the passive listening condition over electrodes Fz, P3 and P4. B depicts differences between SON (solid line) and UN (dashed line) while C shows the different modulation of FV (solid line) and UFV (dashed line). Zero marks the presentation of the stimuli.

Supplementary Table 1. The table depicts individual ERS/ERD values for the active condition. The left columns represent values for alpha ERD in the time window 400-600 ms for midline electrode (Fz, Cz, Pz). In the active condition alpha ERD is present in 81% of the subjects for targets and in 40% for non-targets. The right columns represent values for theta ERS in the time window from 200 to 400 ms for central electrodes (C3-C4). Theta ERS is evident in 100% of the subjects for the target condition and in 92% for the non-target condition. Negative values indicate desynchronization (ERD) with respect to the baseline and asterisks (*) denote significant values for one-sample t-test against zero (i.e., baseline power).

Supplementary Figure 2: Topographic maps of theta ERS in the passive condition (200-400 ms). (A) Theta ERS do not show any significant difference between own name and others name. (B) Stimuli uttered by a familiar vs. unfamiliar voice evoke stronger but not statistically significant theta ERS over the frontal portion of the scalp.
