# Supplementary material for: Oscillatory brain responses to own names uttered by unfamiliar and familiar voices
Source: Brain Res. 2014 Dec 3;1591:63–73. doi: 10.1016/j.brainres.2014.09.074 (PMC4235780; doi:10.1016/j.brainres.2014.09.074)
Supplement: Supplementary file 2 — Supplementary material [file mmc2.docx]

Table S1: Single Subjects ERS/ERD values in the Active Condition

| **Subject** | **Active Condition** | | | | | | | | | | | | |
| --- | --- | --- | --- | --- | --- | --- | --- | --- | --- | --- | --- | --- | --- |
|  | **Alpha 400-600ms** | | |  | **Alpha 400-600ms** | | |  | **Theta 200-400ms** | |  | **Theta 200-400ms** | |
|  | **Target** | | |  | **Non Target** | | |  | **Target** | |  | **Non Target** | |
|  | Fz | Cz | Pz |  | Fz | Cz | Pz |  | C3 | C4 |  | C3 | C4 |
| 1 | -0.60 | 8.64 | -2.08 |  | 5.66* | 6.52* | 0.60 |  | 32.1* | 24.9* |  | 5.31 | -2.44 |
| 2 | -16.5* | -15.1* | -21.9* |  | 0.52 | 9.63 | 2.03 |  | 16.2* | 10.7* |  | 4.84 | -5.08 |
| 3 | -2.12 | 5.03 | 2.17 |  | 2.37 | 12.5 | 18.7* |  | 19.4* | 28.8* |  | 25.7* | 26.5* |
| 4 | -14.1* | -7.32 | -5.00 |  | -1.74 | -13.3* | -10.6* |  | 37.9* | 26.0* |  | 8.57* | 10.2* |
| 5 | 19.7* | 20.7* | 11.82 |  | 0.37 | -3.24 | -5.59 |  | 35.0* | 38.7* |  | 27.9* | 16.2* |
| 6 | -11.5* | -21.2* | -39.8* |  | 12.1 | 12.3* | 11.5 |  | 28.0* | 32.9* |  | 7.90 | 27.7* |
| 7 | 3.85 | -12.7* | -14.0* |  | -13.6 | -16.6* | -16.0* |  | 35.1 | 45.6 |  | 26.5 | 25.0 |
| 8 | -27.7* | -27.1* | -35.7* |  | 6.13 | 1.34 | 19.0 |  | 27.3* | 8.05* |  | 35.4* | 35.1* |
| 9 | -14.4* | -35.2* | -52.6* |  | 3.65* | 4.31 | -10.2 |  | 24.6* | 11.2 |  | 41.9* | 29.6* |
| 10 | -20.8* | -27.9* | -30.0* |  | -1.65 | -2.24 | 0.58 |  | 24.1* | 14.0 |  | 18.3* | 14.8 |
| 11 | 26.1* | -1.48 | -8.16 |  | 7.87 | 5.67 | 13.8 |  | 38.7* | 38.0* |  | 45.5* | 37.9* |
| 12 | -9.65 | -14.9* | -27.9* |  | 1.53 | 5.90 | -14.3* |  | 38.0* | 36.3* |  | 49.6* | 43.5* |
| 13 | -0.59 | -2.48* | -15.3 |  | 1.35 | -7.08 | -8.49 |  |  |  |  |  |  |
| 14 | -15.2* | -12.7* | -16.8* |  | -9.97 | -9.40 | -18.1* |  | 41.6* | 27.7 |  | 13.7* | 11.9 |
