# Supplementary figures and images for: Oscillatory brain responses to own names uttered by unfamiliar and familiar voices
Source: Brain Res. 2014 Dec 3;1591:63–73. doi: 10.1016/j.brainres.2014.09.074 (PMC4235780; doi:10.1016/j.brainres.2014.09.074)

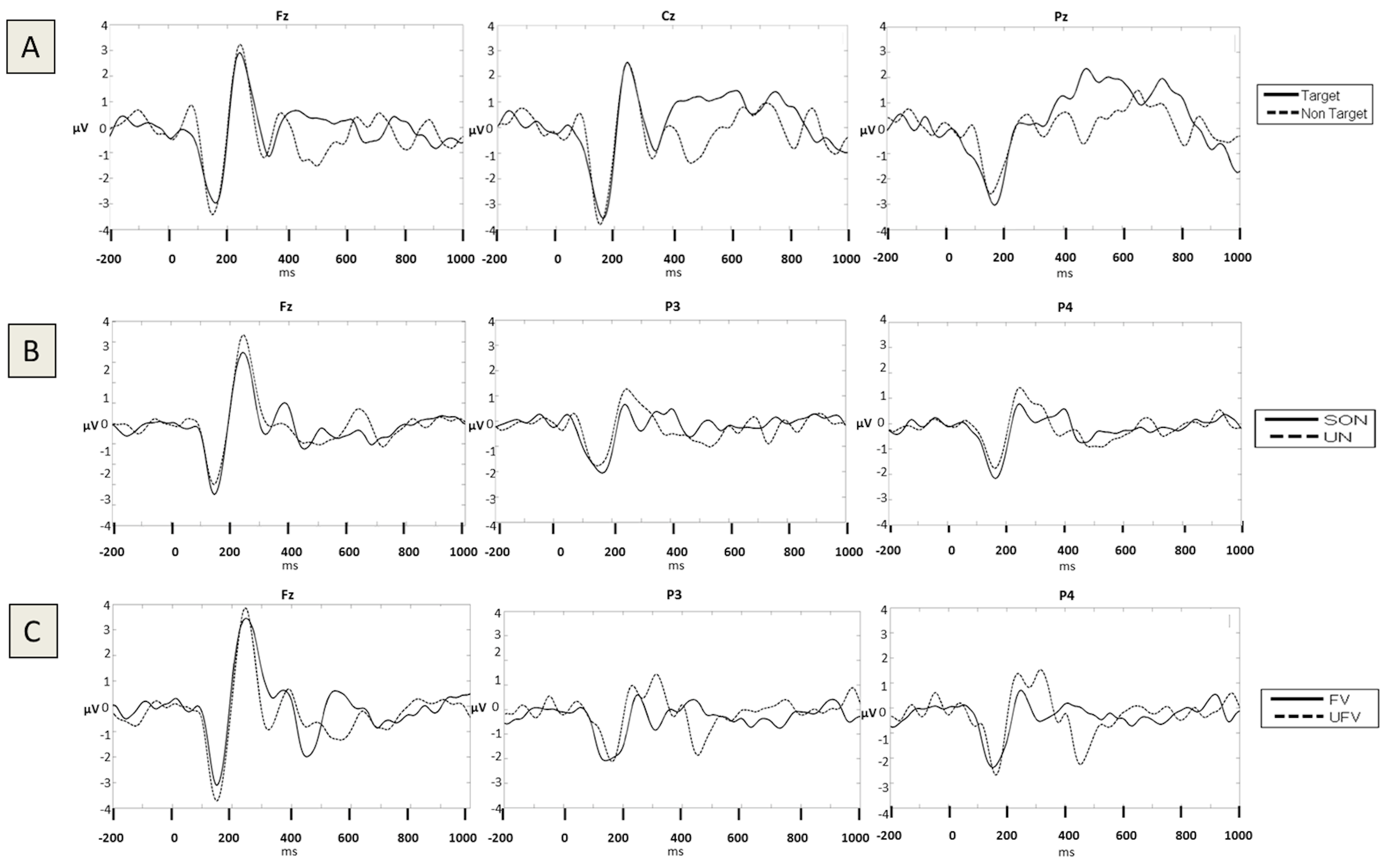

Supplement: Supplementary file 3 — Supplementary material [file mmc3.zip › Suppl.Figure1.tif]

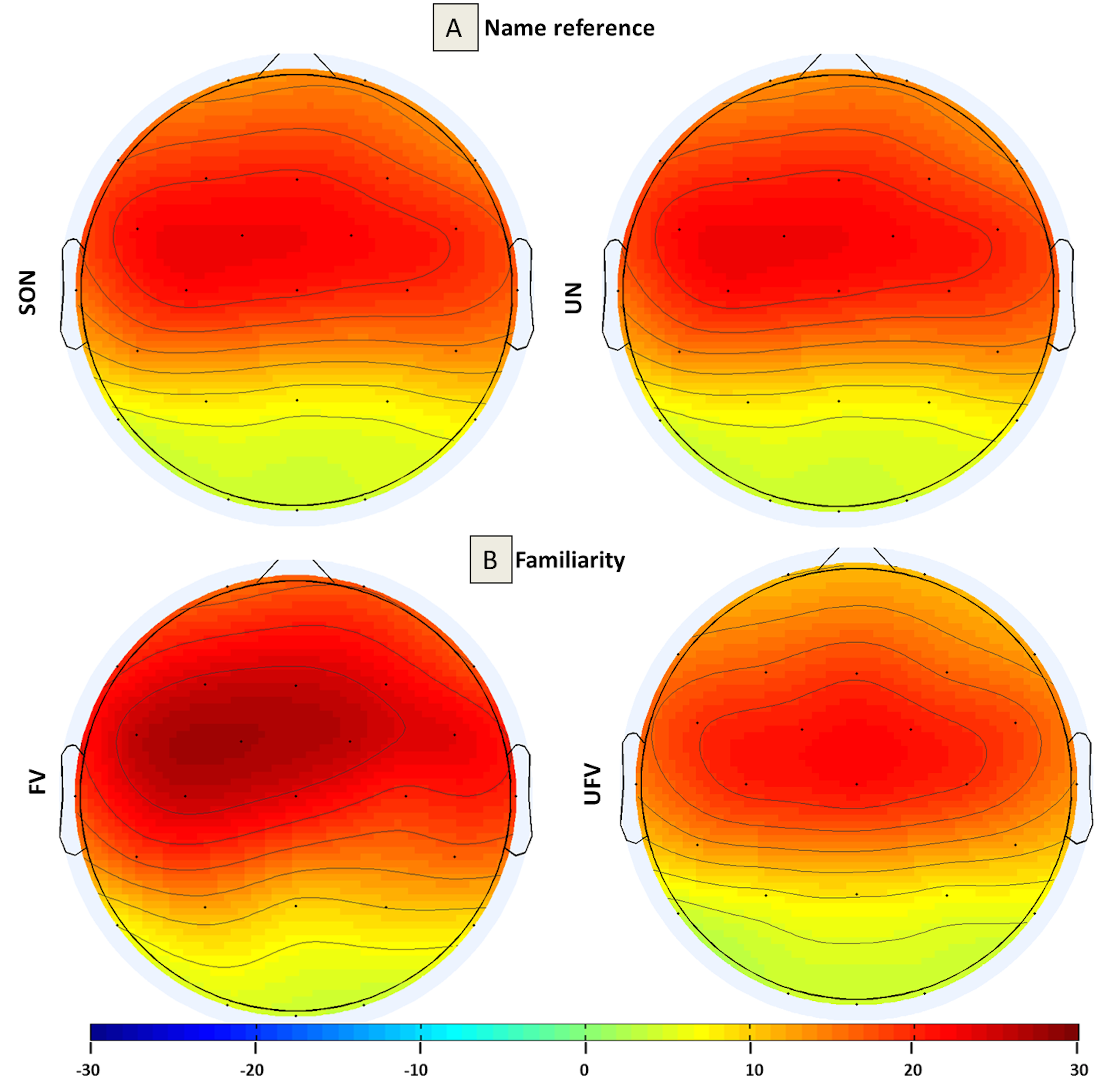

Supplement: Supplementary file 4 — Supplementary material [file mmc4.zip › Suppl.Figure2.tif]
